# Current and future perspectives on the management of polypharmacy

**DOI:** 10.1186/s12875-017-0642-0

**Published:** 2017-06-06

**Authors:** Mariam Molokhia, Azeem Majeed

**Affiliations:** 1 Department of Primary Care and Public Health Sciences, King’s College London, London, SE1 3QD UK; 2 Department of Primary Care and Public Health, Imperial College London, W6 8RP, London, UK

**Keywords:** Polypharmacy, Multimorbidity, Patient safety

## Abstract

**Background:**

Because of ageing populations, the growth in the number of people with multi-morbidity and greater compliance with disease-specific guidelines, polypharmacy is becoming increasingly common. Although the correct drug treatment in patients with complex medical problems can improve clinical outcomes, quality of life and life expectancy, polypharmacy is also associated with an increased risk of adverse drug events, some severe enough to result in hospital admission and even death. Hence, having systems in place to ensure that medications are started only when there is a suitable indication, ensuring patients are fully aware of the benefits and complications that may arise from their treatment, and reviewing patients regularly to ensure their medication regime remains appropriate, are essential.

**Discussion:**

The development and rapid uptake of electronic patient records – particularly in primary care settings where the majority of prescribing takes place – makes monitoring of patients more straightforward than in the past; and allows identification of sub-groups of patients at particularly high risk of adverse drug events and complications. It also facilitates ‘deprescribing’ the process by which medications are reviewed and stopped if not clinically beneficial. In recent years, we have also seen the development of smartphone ‘apps’ to improve communication between patients and healthcare professionals, improve people’s understanding of their conditions and their treatment, and maintain a record of changes made to patient’s medication. In the longer term, developments such as the introduction of artificial intelligence and clinical decision support systems also have the potential to improve prescribing and minimise the risks from polypharmacy. Finally, there is considerable scope to improve the quality of prescribing and reduce risks from poly-pharmacy using non-medical groups such as pharmacists, specialist nurses and physician assistants.

**Summary:**

Polypharmacy has increased in recent decades and will continue to increase as populations age and the number of people with multiple long-term conditions increases. As with all areas of medicine, the evidence-base in this area continues to evolve. Further trials on the impact on patients with polypharmacy of new interventions such as technology-based solutions and the use of different professional groups are needed to improve the evidence-base in this area.

## Background

Polypharmacy is the concurrent use of multiple medications by one individual. It is becoming increasingly common in the United Kingdom and in other countries as populations age, the number of people with long-term conditions rises, and doctors come under increasing pressure to follow evidence-based guidelines for chronic disease management; such as those issued by the National Institute for Health and Care Excellence in England [1]. Traditionally, these guidelines have been based on single diseases and have rarely considered multimorbidity. Inevitably, therefore, older patients who have co-existing medical problems may end being prescribed several different drugs by their physicians. For example, a patient with type 2 diabetes, hypertension and osteoarthritis may be prescribed one or more oral hypoglycaemic agents, an angiotensin converting enzyme (ACE) inhibitor, other anti-hypertensive agents, a statin, aspirin, and an analgesic.

The relative absence of up to date evidence-based guidelines is a key limitation in the management of multimorbidity and its associated polypharmacy. There have been numerous ‘single-disease’ guidelines published by many professional societies and government agencies. Developers of guidelines such as the National Institute for Health and Care Excellence are now recognising this and are developing guidelines that consider the increasing number of people who have more than one significant long-term condition [2]. However, the development of these new guidelines and their impact on clinical outcomes and patient experience will take some time to become apparent because of the lack of clinical trials in patients with multi-morbidity. Randomised clinical trials in the past have often had quite rigorous selection procedures that resulted in older, frailer patients with multi-morbidity being excluded. These however are the patients that are seen increasingly in clinical practice; and who are most likely to be subject to polypharmacy. In the absence of data on these patients from clinical trials, an alternative method of generating evidence about the benefits and risks of polypharmacy is to use data from large clinical databases or patient registries [3].

Although the correct combination of drugs in patients with complex medical problems can improve their health status, clinical condition and quality of life, polypharmacy also increases the risk of drug interactions and side-effects; for example, hyponatraemia or postural hypotension through the use of diuretics or antihypertensive agents. These drug-induced adverse events can sometimes be severe enough to necessitate hospital admission (for example, postural hypotension could result in a fall leading to a fracture) and can occasionally even result in death [4]. Even in milder cases, adverse drug reactions and drug interactions can have a significant effect on a patient’s quality of life. In this article, we will review trends in polypharmacy and how clinicians can try to ensure they maximise the benefits of prescribing and minimise the associated complications; particularly in the increasing number of frail, elderly patients that physicians are now seeing in health systems across the world.

### How do we define polypharmacy?

There is no standard definition of polypharmacy. Although many studies on this topic simply report a count of the number of prescription drugs, this is a crude measure [5]. For example, basing the definition of polypharmacy simply on the number of drugs a patient is receiving does not take into account the benefits that patients may receive from their medication (Table 1). Moreover, the number of drugs that patients receive has been steadily increasing for many years. Hence, a drug count that might have been considered high 20-30 years ago may no longer be considered high now. For example, a study from Scotland reported that the mean number of drugs received by patients increased from 3.3 in 1995 to 4.4 in 2010; and the mean number of drugs received by patients may well have increased still further since then [6].

**Table 1:** Some sources of patient benefits and harms from polypharmacy

Benefits	Outcome
Improved disease management	Reduced risk of disease complications and mortality
Optimised medicines management	Evidence based prescribing
Harms	Consequences
Increased drug interactions	Electrolyte disturbances; potentiation of drug effects
Increased risk adverse drug outcomes	Patient morbidity and mortality
Inappropriate prescribing and medication errors	Unscheduled contacts with healthcare providers
Lack of monitoring	Safety risks to patients

The same study also reported that the number of patient receiving 5 or more medications and 10 or more medications increased by 1.8-fold and 3.1-fold respectively during the study period; and the percentage of people aged 65 and over receiving 10 or more medications had reached 16.4% by 2010. A study from England reported that 17% of patients in primary care were receiving between 5 and 9 medications and an additional 9.7% were receiving 10 or more medications [7]. Studies from other countries have reported similar findings: an increase in prescribing rates over time, as well as an increase in the proportion of patients on 10 or more drugs.

Alternative measures of polypharmacy other than simple counts include the number of potentially inappropriate drugs or drug-combinations, based on pre-defined criteria (such as the widely-used Beers criteria or STOPP/START criteria) [8, 9]. More recently, to help distinguish between ‘good’ and ‘bad’ polypharmacy, the terms “potentially appropriate” and “potentially problematic polypharmacy” have been suggested. Appropriate polypharmacy is defined as prescribing for a patient with complex medical conditions or for multiple conditions in circumstances where medicine use has been optimised or where medicines are prescribed based on the best available evidence. Problematic polypharmacy is defined as prescribing of multiple medications inappropriately or where the patient does not receive the intended benefit of the medication. A key aim of such tools is to identify potentially inappropriate prescribing; particularly in frail, older patients who are the group most at risk of adverse events [10].

### Risks from polypharmacy

At age 65 years, around 8 years of the 20 remaining years of life can be expected to be lived with polypharmacy. After age 75 years, more than half of the remaining life expectancy will be spent with polypharmacy. Higher rates of prescribing are in turn associated with higher rates of potentially unsafe prescribing and rates of adverse events. The PRACtICe Study reported that 30% and 47% of patients receiving 5 or more and 10 or more medications respectively had prescribing or monitoring errors in the 12-month study period [7]. After adjusting for other factors, each additional medication increased the risk of an error occurring by 16%. A Scottish study of patients in primary care thought to be vulnerable to adverse drug events reported that 14% of patients had received a high-risk prescription in the past year. The risk factor most strongly associated with high risk prescribing was the number of drugs prescribed: patients on more than 10 medications had a nearly 3-fold increase in high-risk prescribing compared with those receiving 1 or 2 medications [11].

### High risk groups

The risks from polypharmacy are higher in vulnerable groups, including those with existing co-morbidities such as diabetes and rheumatological diseases, and older patients [12]. Patients living in care homes and housebound patients are also at higher risk of complications from polypharmacy. The complications associated with polypharmacy can include adverse clinical outcomes such as renal failure and falls leading to fractures, as well as an increased risk of mortality [13]. There is also a financial impact on health systems through outcomes such as an increase in urgent hospital admissions and hospital readmissions [14]. In a study of patients with rheumatoid arthritis, increasing drug use was associated with more frequent acute hospitalisations; patients in the highest drug use group (≥10 drugs) had a more than 3-fold increase in the rate of hospitalisation compared to those in the lowest drug use group (0-5), even after adjustment for age and sex. The risk of hospitalisation was even higher in those taking steroids [15]. In developed countries, older people will form an increasingly large proportion of the population and this ‘aging’ of the population will be associated with a parallel increase in the number of people with long-term conditions such as hypertension, arthritis, diabetes and heart disease. Hence, polypharmacy in at risk groups - and the elderly in particular - will become an increasingly important issue for patients, carers, clinicians, health systems and societies.

### Identifying patients with polypharmacy

Duerden and Avery, in their report for the King’s Fund, outline a pragmatic approach to identifying patients with polypharmacy and identifying ‘at risk’ patients using a combination of patient characteristics and the number of drugs prescribed [10]. This approach is based on prior research showing an association between adverse health outcomes and polypharmacy, and that this association is more marked in patients with major illnesses. Duerden and Avery recommend focusing on patients who are on 10 or more drugs; or patients receiving 5-9 drugs who have other risk factors such as a major comorbidity (e.g. diabetes or rheumatoid arthritis), have suffered previous adverse drug reaction, or are from a vulnerable group (e.g. people living in care homes or with a learning disability). Another UK study reported that the three commonest drugs linked to adverse drug reactions that resulted in hospital admission were non-steroidal anti-inflammatory agents, diuretics, and warfarin [16]. Studies such as this can guide clinicians as to which patients to focus on so they can identify those who may be at highest risk from the complications associated with polypharmacy.

The rapid development, implementation and use of electronic patient records in primary care greatly simplifies the process of identifying patients with polypharmacy. In the United Kingdom for example, there is now 100% uptake of electronic patient records in general practice, with high rates of use also seen in many other developed countries. Searches to identify patients with polypharmacy that might have taken hours (or even days) to complete with paper-based records can now be carried out in minutes. As well as identifying patients based on the number of drugs they are taking, it is straightforward to also include variables such as age, drug group or laboratory test results into such searches. This can allow physicians to identify high risk groups who would benefit from medication reviews and closer monitoring of their prescribing and their illnesses (for example, those or warfarin or with raised creatinine levels). In the future, linkage of primary care records with hospital admission records can further improve this process by allowing the identification of individuals who have had an acute hospital admission from an adverse drug reaction or a drug interaction.

### Managing polypharmacy in patients with multimorbidity

In 2016. England’s National Institute for Health and Care Excellence published guidance on the clinical assessment and management of patients with multimorbidity (defined as the presence of two or more long-term health conditions). The guidance emphasizes the need to deliver care in a way that considers multimorbidity and any associated frailty in patients. This includes, for example factors, such as:

- how the person’s health conditions and their treatments interact and how this affects quality of life
- the person’s individual needs, preferences for treatments, health priorities, lifestyle and goals
- the benefits and risks of following recommendations from guidance on single health conditions
- improving quality of life by reducing treatment burden, adverse events, and episodes of unplanned care

The NICE guidance recommends that clinicians consider evidence of likely benefits and harms for the individual patient and outcomes important to the patient. This could be for example through the use of a screening tool (for example, the STOPP/START tool in older people) to identify medicine-related safety concerns and medicines the person might benefit from but is not currently taking. The important of reviewing patients to monitor the effects of any changes made to prescribing are also emphasised, which would include the need to whether any further changes to treatments are needed (including restarting a treatment). The importance of shared decision-making is also a key part of this guidance; this is particularly important in people with frailty or limited life expectancy who have less capacity to benefit from pharmacological interventions.

### Risk prediction tools

One method of facilitating guidance on managing patients with polypharmacy would be through the development and application ‘risk prediction tools’ for quantifying the risk of adverse drug reactions. A systematic review published in 2014 evaluated the quality of validated risk-prediction tools for adverse drug reactions in people over 65 years of age [17]. The authors of the review identified four main tools [18–21]. However, all the risk prediction tools had limitations and hence their performance was generally modest [17]. In addition to their relatively weak performance, these tools were all developed using data for hospital inpatients and we do not therefore know how well they would perform for patients in ambulatory or primary care settings. Further research in this area should therefore focus on developing risk prediction models that can be used in out-of-hospital settings as this is where the majority of prescribing for patients with long-term conditions takes place. Another key finding from this review is that many variables contribute to the risk of adverse drug reactions and it is therefore difficult to develop robust risk prediction tools. Hence, although several risk tools exist, none currently have sufficient predictive value for use in routine clinical practice [22]. Consequently, it may therefore be some time before we risk scores for patients with polypharmacy that are for example as widely used as tools for assessing cardiovascular risk, such as QRISK in the UK [23, 24].

The use of electronic patient records for ‘secondary’ uses such as this that are not directly for the care of an individual patient does bring ethical, political and technical challenges. In England, the government announced in July 2016 that the care.data programme that was designed to harness the potential of the electronic patient records that the NHS holds for work like this was to be abandoned [25]. The care.data programme – although it promised many benefits for research and quality improvement – ran into considerable opposition from clinicians, patient groups and the public because of concerns about the ethics of ‘harvesting’ data from people’s electronic health records without their explicit consent; and because of the potential security issues and threats to confidentiality from storing such a large amount of personal data in one place. The key lessons from this episode for health systems in other countries are that patients and the public need to be convinced about the benefits of use of their data for secondary purposes not directly related to their clinical care; and that smaller, less ambitious, more targeted projects in this area are more likely to receive public support and to be implemented.

### Evidence base on optimising prescribing

Two systematic reviews are relevant here, one which looked at improving outcomes for people with multiple chronic conditions’ and the other at improving the appropriate use of polypharmacy in older people. Smith et al. identified 18 relevant randomised controlled trials of interventions that aimed to improve the management of people with multimorbidity and common comorbidities in primary care and community settings [26]. They reported that there was a lack of evidence about the effectiveness of interventions for people with multimorbidity because of the relatively small number of trials conducted on this topic thus far, and that the trials that had been carried out has mixed findings. There was some evidence though of improved health outcomes if interventions can be targeted at risk factors such as depression or specific functional difficulties in people with multimorbidity. Cooper et al. identified 12 studies (of which 10 were randomised controlled trials) of interventions that aimed improve the use of polypharmacy in older people [27]. There were a range of interventional strategies deployed and some evidence that these led to more appropriate polypharmacy (based on lower levels of inappropriate prescribing). However, it was not clear if these interventions led to clinically important improvements (for example, a reduction in emergency hospital admissions).

### Optimising the use of medicines

Given the risks from polypharmacy, improving the use of medicines – and reducing the risks to patients from inappropriate or unsafe prescribing – should be a priority for health systems [28]. All prescribers need to carefully consider the potentials costs as well as the benefits of treatment before starting a drug; and be aware of the risks of over-treatment, drug interactions and adverse drug reactions. Until recently, health systems and professional societies have focused on producing single-disease guidelines and we are only now seeing some progress in developing guidelines for managing patients with multimorbidity. Tools to promote shared decision-making by doctors and patients are also a relatively new development. In recent years, we have seen the introduction of initiatives such as the International Patient Decision Aids (IPDAS) collaboration, the Dutch Decision Aids Implementation Programme and the Center for Informed Choice in the USA [29].

Prescribers also need to have systems in place for monitoring prescribing and reviewing patients regularly. The use of electronic patient records can facilitate this monitoring, as can the use of other professional groups such as pharmacists, specialist nurses and physician assistants to support doctors in this monitoring and reviewing role. In England, the General Practice Forward View aims to provide investment to recruit such non-medical professional groups to support general practitioners, thereby freeing up physicians’ time to focus on more complex patients [30].

An intervention using data from linked electronic medical records showed that intervening in primary care practices can significantly reduce rates of high-risk prescribing of drugs [28]. The study, which was carried out 33 medical practices with a registered population of around 200,000 patients in the Tayside region of Scotland, also showed that the change in prescribing patterns can lead to significant reductions in related emergency admissions to hospital. The study team examined patients’ exposure to high-risk prescribing of non-steroidal anti-inflammatory drugs or antiplatelet agents. This included prescriptions to people with kidney disease or heart failure, or prescribing to people taking anticoagulant drugs like warfarin. The 48-week intervention comprised professional education, informatics to facilitate reviews of patient treatment, and small financial incentives for practices to review patients. The interventions led to a 37% reduction in high-risk prescribing, and this improvement was sustained after financial incentives to review patients were withdrawn. There was also an associated reduction the rate of hospital admissions for gastrointestinal ulcer or bleeding, and for heart failure.

### Vulnerable populations

People living in care homes are perhaps the frailest and most vulnerable group in the community. They are particularly likely to be on multiple prescription medications and are also at high risk of complications from inappropriate prescribing. A systematic review of studies that aimed to improve prescribing for people living in care homes concluded that there is no one interventional strategy that has proved to be effective [31]. The interventions examined included staff education, pharmacist-led medication reviews, multi-disciplinary team meetings, and computerised clinical decision support systems. There was some evidence that a multi-faceted approach to optimising prescribing that used more than one intervention was more likely to be successful than a single intervention. Given the lack of evidence, it is clear that this is an area that would benefit from additional research. In particular, the use of computerised clinical decision support systems that can use the information held in electronic patient records is an avenue that should be explored. However, thus far, computerised decision support systems have provided only limited evidence of benefit and considerable more developmental work needs to be done to harness their potential [32, 33]. Another systematic review of interventions targeting older adults living in the community reached similar conclusions. The interventions examined in the review were organizational (pharmacist interventions), professional (computerized clinical decision support systems), and multifaceted approaches. The interventions did appear beneficial in reducing potentially inappropriate prescribing, but the range of effect sizes was modest, and it is unclear whether such interventions can result in clinically significant improvements in patient outcomes. As with many such studies, the authors concluded this was an area where more research was needed [34].

### Artificial intelligence and cognitive computing

In the longer-term, it is possible that we could see the use of automated analytical techniques, artificial intelligence technologies and ‘deep learning methods’ such as those developed by companies such as DeepMind could also be applied in this area [35]. One example of this is research in this area is a project to improve the detection of acute kidney injury, many cases of which are drug-induced, and which is a major cause of morbidity and mortality globally. Using techniques to integrate and analyse clinical data from a range of sources, DeepMind aims to automatically identify patients with acute kidney injury and notify their clinicians promptly; thereby aiming to detect acute kidney injury earlier and giving clinicians the opportunity to change a patient’s clinical management [36]. Similar methods could also be used to risk stratify patients to identify those at higher risk of complications, thus giving their clinical teams the option to modify their patients’ treatment to reduce this excess risk [37]. A related area is the development of ‘cognitive computing’. This is being pioneered by companies such as IBM, which is using its Watson Health Platform to support clinicians to optimise treatment and prescribing decisions for patients. The Watson platform is based on natural language processing and machine learning from very high volumes of unstructured clinical data. Although technologies such as artificial intelligence and cognitive computing are still in a very early phase of development, considerable financial investments are being made in this field by companies such as Google and IBM. It seems inevitable therefore that in future years such technologies will become increasingly commonplace in healthcare settings, radically changing the way in which doctors and other health professionals work; as well as giving patients more support in the management of their long-term conditions and in optimising their health [38].

### Medicine reconciliation

Another key area in polypharmacy is ‘medicines reconciliation’: ensuring that when patients are discharged from hospital, particularly after an acute admission, systems are in place for rapidly communicating any changes in medication, and why these changes took place, to primary care teams. Prior research has shown that the discharge summaries received by primary care teams are often inaccurate or lacking key information; and this is an area where better collaboration is needed between primary care and secondary care teams to develop safer discharge policies and reduce the risk of medication-related errors. A systematic review and meta-analysis of medicines reconciliation published in 2016 showed that medicines reconciliation can reduce adverse drug event-related attendances at emergency departments and readmissions to hospital [39].

Another key step in reducing risks from polypharmacy is to improve the discharge process for patients [40, 41]. This would include:

- Ensuring that discharge arrangements are discussed with patients, family members and carers; and that they are given a copy of the discharge summary.
- Adequate coordination between the hospital, community health services, general practices, and the providers of social care services.
- There is a follow-up after discharge of patients at high risk of complications or readmission - either in person or by telephone - to ensure that the discharge arrangements are working well.
- Medicines reconciliation is carried out. This is the process of verifying patient medication lists at a point-of-care transition, such as hospital discharge, to identify which medications have been added, discontinued, or changed from pre-admission medication lists.
- Ensuring that any outstanding test results at discharge are obtained and passed on to primary care teams; and ensuring there are clear arrangements for carrying out and acting on any proposed post-discharge tests.

### Management of side effects

Side effects from drugs are common, with the highest rates seen in patients with polypharmacy. Previous research has shown that many patients do not report the side effects of their drugs to their physicians; and when they do inform their physicians, these side effects are sometimes not recorded in patients’ medical records or reported to regulatory authorities. Prior research has shown that physicians prescribing new medications often do not convey important medication-related information to their patients and this is an area of practice that needs to be improved. A physician-targeted educational session in the USA improved the content of and enhanced patient ratings of physician communication about new medication prescriptions [42]. Education of patients in turn can improve their reporting of side effects to their physicians. New smartphone applications aimed at patients with long-term conditions have the potential to help in the recording and management of drug side-effects but their use requires further evaluation [43]; as do systems for allowing patients to self-report drug side effects to national pharmaceutical regulatory organisations [44].

### Involving patients

A key component of addressing the risks associated with polypharmacy is to ensure that patients are fully involved in the decision to start a drug; and also in monitoring their use of medication to ensure appropriate adherence to their prescribed drug regime. This will include briefing patients about the risks of their medication, as well as its benefits; the importance of undergoing a regular medication review; reporting any adverse events promptly to their physician; and discussing with patients ‘reminder’ systems such as dosette boxes to ensure they take their medication at the right time and dosage. The use of polypills that allow patients to take one pill in place of several could also improve patients’ use of their medication [45].

One negative effect of giving patients ‘too much’ information however is that that they may then be discouraged from taking a drug because of concerns about its side-effects. Tools to promote shared decision-making can help overcome such concerns and improve patients’ adherence to their proposed management plan by providing information on the benefits and risks of drugs in a format patients can understand more easily [46]. Awareness of the relationship between patients, their social networks, and health services as in the ‘Burden of Treatment’ model described by May and colleagues can also help approve patient’s adherence to their medication [47].

Another area where shared decision making can help in is ‘deprescribing’, the process of planned and supervised tapering or ceasing of inappropriate medicines [48]. Deprescribing is particularly important in frail, older patients or in patients with limited life expectancy in whom the inappropriate use of medication is widespread, and who are at high risk of adverse drug reactions, hospitalisation and death. Optimization of medication in these patient groups can be challenging but can yield considerable benefits – for both patients and health systems – through appropriate use of deprescribing. Through a review of previously proposed deprescribing processes, Reeve and colleagues developed a patient-centred deprescribing process, using a five-step cycle that included a comprehensive medication history, identifying potentially inappropriate medications, determining whether the potentially inappropriate medication can be stopped, planning the withdrawal process; along with provision of monitoring, support and documentation.[Reeve] This approach shows promise and now needs to be tested in a wider range of clinical settings.

In recent years, we have also seen the development of smartphone ‘apps’ such as the My Medication Passport to improve communication between patients and healthcare professionals, improve people’s understanding of their conditions and their treatment, and maintain a record of changes made to patient’s medication [49]. These smartphone-based tools do show promise – as does giving patients online access to their electronic medical records - but such technologically-based interventions need rigorous evaluation to determine if clinicians, patients and carers will use them, and to ensure that they are cost-effective [50]. For example, a review of smartphone apps for patients with asthma found a number of errors in many of them and a lack of compliance with current clinical guidelines [43].

One important limitation of the use of information technology based tools is that older patients – who are the main target for interventions to improve prescribing - are the least likely to use them. This will change over time as devices such as smartphones become more widely used but for now, clinicians will also have to use paper-based tools for such patients if they are unwilling or uncomfortable with using information technology-based tools. We should also not also overlook that work to improve prescribing regimes and reduce the problems associated with inappropriate polypharmacy will require considerable levels of inter-professional working, support from patients; and substantial behavioural change from both clinicians and patients.

### Personalised medicine

The key message from prior research on polypharmacy is the need to manage the risks and benefits of drug treatment. This is an area where ‘personalised medicine’ approaches may help. In recent decades, healthcare has gradually progressed from being reactive to care that is predictive, preventive, personalized and participatory [51]. This new approach to healthcare will eventually provide patients and physicians with personalised information about each individual’s unique health experience. This information can help personalise care to each person’s unique characteristics. This approach can be further enhanced through support from ‘digital patient communities’ that allow patients to interact with other patients with similar long-term conditions and learn from the experience of other patients [52]. Such digital communities are now common for many conditions and are a particularly useful source of support for those patients with very rare conditions.

## Conclusions

Polypharmacy, however it is defined, has increased in recent decades and will continue to increase as populations age and the number of people with long-term conditions also increases. For high risk individuals and drug classes, targeted reviews and appropriate regular monitoring can help address the increased risks of adverse events from polypharmacy. Non-medical professionals such as pharmacists, specialist nurses and physician assistants have a key role to play in this area as does making full use of the benefits offered by electronic patient records. Clinicians also need to ensure that patients are fully involved in decisions about their prescribing and in the monitoring of their medical problems. Simple aids such as dosette boxes can help patients use their medication appropriately.

The development of smartphone apps, clinical-decision support systems using newly-developed artificial intelligence technology, and giving patients online access to their electronic medical records, also show promise to improve the safety and appropriateness of prescribing, and improve patients’ knowledge of their medication. In this era of personalised medicines, we should aim to balance individual risks and benefits for our patients using all the tools available to us; and implement appropriate mechanisms for monitoring and reviewing prescribing decisions in our patients. As with all areas of medicine, the evidence-base in this area has many gaps and continues to evolve. Further trials on the impact on patients with polypharmacy of new interventions such as technological solutions and the use of different professional groups and multi-disciplinary working are therefore needed to give patients and clinicians the information they need to make full-informed decisions.
